# Notes and News

**Published:** 1897-03-06

**Authors:** 


### NOTES AND NEWS.

(Collected and Communicated.)

HOSPITAL MEETINGS.

Dental Hospital of London, Leicester Square.—Annual general meeting, Thursday, March 11th, half-past five, at the Hospital.

Hospital for Diseases of the Throat, Golden Square.—Annual general meeting at 20, Hanover Square, Monday, March 15th, half-past three.

Mr. Mewburn is providing a children's and isolation ward and a new kitchen at the Horton Infirmary.

Lord Wimborne has offered Poole Mansions as a hospital for Poole and the neighbourhood, and has offered £1,000 towards its endowment.

Mr. Alfred Willett has been appointed Bradshaw Lecturer for the present year at the Royal College of Surgeons.

Sir John Conroy has accepted the office of treasurer to the Radcliffe Infirmary, Oxford, in place of Lord Dillon, resigned.

The foundation-stone of a new infirmary in connection with the St. Helen's Workhouse has just been laid. The total provision will be for 350 beds, and the cost is estimated at £20,000.

Mr. Sholto Hare has offered the Almondsbury Cottage Hospital to Bristol. The institution was opened in 1892 in memory of Mrs. Sholto Hare. It contains three wards for five patients and a convalescent ward. The structure is handsome, and is well-fitted and furnished.

At a special meeting of the staff of the Medical School of Guy's Hospital, held on Thursday, February 18th, 1897, it was unanimously resolved: "That the physicians, surgeons, and lecturers of Guy's Hospital desire to express to Her Majesty's Government their earnest hope that a measure may be introduced into Parliament this Session to appoint a Statutory Commission for the reconstruction of the University of London, in accordance with the report of Lord Cowper's Commission."

The Mansion House Fund for the Relief of Sufferers from Famine in India now reaches £378,000.

The council of the Sanitary Institute have accepted an invitation from the city of Leeds to hold a congress and health exhibition there in September next.

At a meeting of the shareholders of the Great Northern Railway of Ireland a proposal to contribute £2,000 to the new Victoria Hospital at Belfast was made. A large number of shareholders opposed the motion, which was consequently not carried.

Sir Thomas Storey has given £500 and Lord Aston £1,300 to wipe off the deficit on the building and furnishing fund of the Royal Lancaster Infirmary. At the meeting of the institution at which this announcement was made Sir Thomas Storey suggested that 5,000 inhabitants of Lancaster might contribute one penny weekly to the infirmary and thus provide the additional income necessary.

Germany has appointed a Commission for the study of the plague as now present in India. The members of the Commission include Professors Koch, Pfeiffer, and Gaffky. Professor Koch will join the Commission on the completion of his researches in Africa. Professor Gaffky, who will be in command until Dr. Koch's arrival, has worked with him previously in India on the same subject.

The New General Hospital for Birmingham is fast progressing towards completion. It had been hoped that the Queen would have been able to perform the opening ceremony, but she has been obliged to decline encountering the unavoidable fatigue. The Princess Christian has kindly consented to act on her behalf, and the opening will probably take place in June. About £45,000 is still required to open the hospital free of debt. Mr. J. Horder has again come forward with a munificent gift, and heads the latest subscription list with the sum of £5,000.

The Macclesfield General Infirmary is about to adopt new rules. It is proposed that in future ten or more subscribers at the annual meeting in February of any year shall have power to alter, make, or repeal rules subject to one month's notice being given to the president of the governing body on the part of the subscriber intending to move such alteration. It is further proposed that the report of the infirmary shall contain a list of the actual and possible attendances of each member of the governing body, and that it shall give an abstract of the proceedings of the governors. To the senior house surgeon is to be allotted, amongst other things, the task of seeing that the nurses properly discharge their duties in their wards, and reporting weekly to the House Committee the general state of the institution. The matron's duties are to be the care of the household goods, housekeeping and domestic management, together with the duty of engaging, suspending, and discharging the domestic servants. She is also to see that the patients have their meals at the appointed times and such as the doctors order.

